# Targeting ASIC3 for Relieving Mice Fibromyalgia Pain: Roles of Electroacupuncture, Opioid, and Adenosine

**DOI:** 10.1038/srep46663

**Published:** 2017-04-25

**Authors:** Liang-Ta Yen, Ching-Liang Hsieh, Hsin-Cheng Hsu, Yi-Wen Lin

**Affiliations:** 1 College of Chinese Medicine, Graduate Institute of Acupuncture Science, China Medical University, Taichung, 40402, Taiwan; 2 College of Chinese Medicine, Graduate Institute of Integrated Medicine, China Medical University, Taichung, 40402, Taiwan; 3 China Medical University Hospital, Department of Chinese Medicine, Taichung, 40402, Taiwan; 4 College of Chinese Medicine, School of Post-Baccalaureate Chinese Medicine, China Medical University, Taichung, 40402, Taiwan; 5 College of Chinese Medicine, Master’s Program for Traditional Chinese Veterinary Medicine, China Medical University, Taichung, 40402, Taiwan; 6 Research Center for Chinese Medicine & Acupuncture, China Medical University, Taichung, 40402, Taiwan

## Abstract

Many scientists are seeking better therapies for treating fibromyalgia (FM) pain. We used a mouse model of FM to determine if ASIC3 and its relevant signaling pathway participated in FM pain. We demonstrated that FM-induced mechanical hyperalgesia was attenuated by electroacupuncture (EA). The decrease in fatigue-induced lower motor function in FM mice was also reversed by EA. These EA-based effects were abolished by the opioid receptor antagonist naloxone and the adenosine A1 receptor antagonist rolofylline. Administration of opioid receptor agonist endomorphin (EM) or adenosine A1 receptor agonist N^6^-cyclopentyladenosine (CPA) has similar results to EA. Similar results were also observed in ASIC3^−/−^ or ASIC3 antagonist (APETx2) injected mice. Using western blotting, we determined that pPKA, pPI3K, and pERK were increased during a dual acidic injection priming period. Nociceptive receptors, such as ASIC3, Nav1.7, and Nav1.8, were upregulated in the dorsal root ganglion (DRG) and spinal cord (SC) of FM mice. Furthermore, pPKA, pPI3K, and pERK were increased in the central thalamus. These aforementioned mechanisms were completely abolished in ASIC3 knockout mice. Electrophysiological results also indicated that acid potentiated Nav currents through ASIC3 and ERK pathway. Our results highlight the crucial role of ASIC3-mediated mechanisms in the treatment of FM-induced mechanical hyperalgesia.

Fibromyalgia (FM) pain, which is a crucial worldwide health problem, is characterized by widespread mechanical pain^1^,^2^. Pregabalin is a presynaptic voltage-gated calcium channel blocker that reduces FM pain and sleep disturbances^3^,^4^. Duloxetine attenuates FM-related pain symptoms but has serious side effects^5^. Milnacipran is a serotonin-norepinephrine reuptake inhibitor (SNRI) designed to reduce the FM symptom of nausea^6^. Repeated acidic saline injections into the gastrocnemius muscle (GM) induce widespread chronic mechanical hyperalgesia that can be reversed by electroacupuncture (EA)^7^,^8^. FM pain in mice can be attenuated by the administration of μ-/δ-opioid receptor agonists^9^ or glutamate receptor antagonists in the spinal cord^10^. The decreased local tissue pH reported in FM activates peripheral nociceptive terminals^7^, and recordings of peripheral dorsal root ganglion (DRG) neurons have shown that lower pH conditions reliably induce inward currents that regulate voltage-gated sodium channel (Nav) functions^11^,^12^,^13^.

Acid-sensing ion channel 3 (ASIC3), the most sensitive ion channel for acid sensation, is mainly localized in peripheral sensory neurons, especially the DRG, and typically associated with several pain conditions^14^,^15^. ASIC3 can be activated by acidic pH (~6.8) and enhanced by lactate^16^,^17^. There are four genes that encode at least seven subtypes of receptors: ASIC1a, ASIC1b, ASIC2a, ASIC2b, ASIC3, ASIC4, and ASIC5^18^,^19^. A recent study showed a reduction in the development of mechanical hypersensitivity after an acid injection in the gastrocnemius muscle of ASIC3-null mice^20^. Another paper showed that the hypersensitivity induced by an intramuscular acidic saline injection was mediated by ASIC3^21^.

Navs are involved in inflammatory pain, as evidenced by the significant amelioration of pain by sodium channel blockers^22^,^23^,^24^. Nav1.7 and Nav1.8 are expressed in the peripheral DRG and responsible for pain conduction^25^. Animal studies showed that Nav1.3, Nav1.7, and Nav1.8 play prominent roles in inflammatory pain and are potentiated by the microinjection of carrageenan and CFA^23^. Hyperalgesia was accompanied by alterations in TTX-sensitive and TTX-resistant Na currents^26^,^27^. CFA-induced inflammation was associated with an increase in Nav1.8 expressions in the DRG that was inhibited by ibuprofen^28^. Persistent hyperalgesia induced by injections of prostaglandin E2 (PGE2) was also accompanied by an increase in Nav1.8 in the peripheral DRG^29^. Therefore, Nav1.8 is the main target for a diverse array of inflammatory mediators that act through a number of second-messenger pathways, including PKA^26^,^28^, PKC^26^,^30^, and extracellular signal-regulated kinase^26^,^31^.

Several molecules, such as N-methyl-D-aspartate receptors (NMDARs), ASIC3, TRPV1, calcium channels (Cav), and substance P (SP), have been implicated in mouse models of FM pain^12^,^13^,^21^,^32^,^33^. Dual acidic saline injections increase the cAMP pathway at the central spinal cord level^34^. Activation of pERK was detected in the anterior paraventricular nucleus of the thalamus and amygdala^33^,^35^. The administration of neurotrophin-3 alleviates acid-induced chronic muscle pain, which is similar to clinical observations^36^. The calcium channel antagonist pregabalin and the M-type voltage-gated potassium channel activator flupirtine were reported to be effective at treating muscle pain^37^,^38^. Acupuncture has been used to control body weight^39^, reduce epilepsy^40^, ameliorate learning and memory impairments^41^, and control pain^26^,^42^,^43^. The analgesic effect of acupuncture activates the release of endogenous opiates^44^ and adenosine^45^.

Our rational is that EA could reduce FM symptoms through ASIC3 receptor and related mechanisms. We hypothesized that EA could attenuate mechanical hyperalgesia by releasing endomorphin or adenosine to inhibit ASIC3 receptor in this FM mouse model. Our data showed that pPKA/pPI3K-pERK signaling pathway was crucial for FM priming in mice. EA at acupoint ST36 reliably reduced dual acidic saline-induced mechanical hyperalgesia and motor dysfunction via ASIC3-Nav1.7/Nav1.8 signaling pathways. We also determined that mechanical hyperalgesia and nociceptive signaling were abolished in ASIC3 knockout mice. This study provides novel and detailed molecular mechanisms behind the use of EA to treat FM-related mechanical hyperalgesia.

## Results

### EA-mediated attenuation of ASIC3 signaling reliably reduces mechanical hyperalgesia through peripheral opioid and adenosine pathways in an FM mouse model

**Figure 1:**
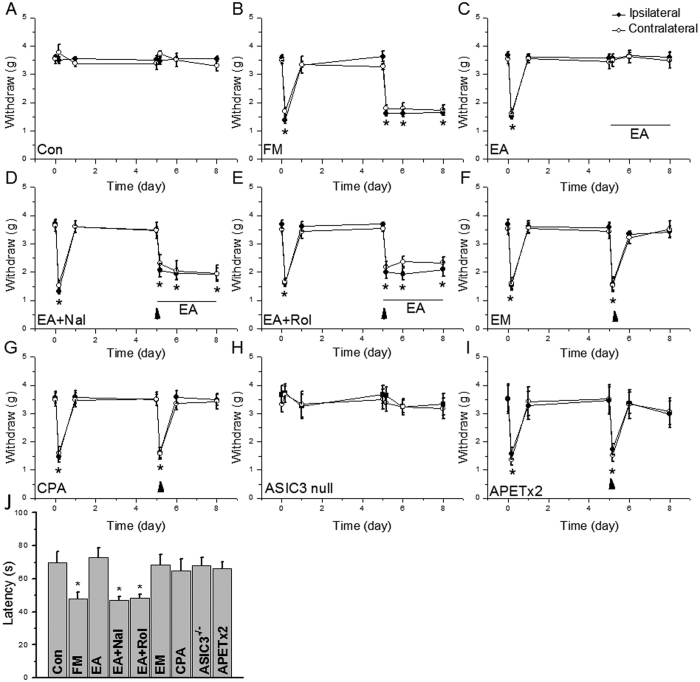
Electroacupuncture (EA) reduced mechanical hyperalgesia induced by dual acid saline injection (fibromyalgia model, FM) as measured by von Frey filaments. (**A**) Mechanical responses of saline-injected control mice. (**B**) Mechanical responses of FM group mice. (**C**) Mechanical responses of FM mice treated with EA (EA group). (**D**) Mechanical responses of FM mice treated with EA and naloxone (Nal group). (**E**) Mechanical responses of FM mice treated with EA and rolofyllin (Rol group). (**F**) Mechanical responses of FM mice treated with endomorphin (EM group). (**G**) Mechanical responses of FM mice treated with EA and N^6^-cyclopentyladenosine (CPA group). (**H**) Mechanical responses of FM in ASIC null mice (ASIC3 null group). (**I**) Mechanical responses of FM mice treated with APETx2 (APETx2 group). Mice were tested before injection (day 0), 4 hours after injection, day 1 (D1), day 5 (D5), day 6 (D6), and day 8 (D8). *p < 0.05 compared to baseline (n = 10 mice per group).

Similar to our previous publication^7^,^12^, two normal saline injections in mice did not induce ipsilateral mechanical hyperalgesia (Fig. 1A, n = 10). Dual intramuscular injections of an acidic solution (pH 4.0) into the gastrocnemius muscle (GM) of mice induced bilateral mechanical hyperalgesia on Days 5–8 (Fig. 1B, n = 10). The first acidic saline injection initiated a rapid and transient mechanical hyperalgesia that recovered after 1 day. A second acidic saline injection 5 days after the first injection induced significant and long-lasting mechanical hyperalgesia. EA at acupoint ST36 for 4 continuous days significantly reduced mechanical hyperalgesia (Fig. 1C, n = 10). To elucidate how EA affects neurotransmitters to attenuate mechanical hyperalgesia, we injected opioid and adenosine antagonists into the acupoint. We showed that the analgesic effect of EA was blocked by the opioid antagonist naloxone (Fig. 1D, n = 10) and adenosine antagonist rolofylline (Fig. 1E, n = 10). Injection of endomorphin (EM) or N^6^-cyclopentyladenosine (CPA) significantly attenuated mechanical hyperalgesia (Fig. 1F and G, n = 10). Mice lacking the ASIC3 gene (*Asic*3^−/−^) showed a bilateral decrease in mechanical hyperalgesia (Fig. 1H, n = 10). Similar results were also observed in ASIC3 antagonist (APETx2) injection (Fig. 1H, n = 10). Additionally, we utilized a rotarod to test fatigue behavior. Our data indicated that lower motor function was observed in FM mice (Fig. 1J, 47.8 ± 3.9 s, *p* < 0.05 compared with Control group, n = 10), and this symptom was alleviated by EA manipulation (Fig. 1J, 73.1 ± 5.6 s, *p* < 0.05 compared with FM group, n = 10). Furthermore, this phenomenon was blocked by naloxone (Fig. 1J, 47.0 ± 2.1 s, *p* < 0.05 compared with Con group, n = 10) and rolofylline (Fig. 1J, 48.1 ± 2.4 s, *p* < 0.05 compared with Con group, n = 10). Injection of endomorphin (EM) (Fig. 1J, 68.3 ± 6.3 s, *p* > 0.05 compared with Con group, n = 10) or N^6^-cyclopentyladenosine (CPA) (Fig. 1J, 64.8 ± 7.2 s, *p* > 0.05 compared with Con group, n = 10) reliably improve motor function. Similar data was also observed in ASIC3^−/−^ or APETx2 injected mice (Fig. 1J, 68.1 ± 4.7 s vs. 66.2 ± 3.9 s, *p* > 0.05 compared with Con group, n = 10). These data suggest that FM-induced mechanical hyperalgesia and fatigue depend on ASIC3, opioid and adenosine receptors.

### Acidic saline injections transiently increased the pPKA, pPI3K, and pERK signaling pathway to deliver nociceptive signals, and the effect is attenuated by EA treatment

**Figure 2:**
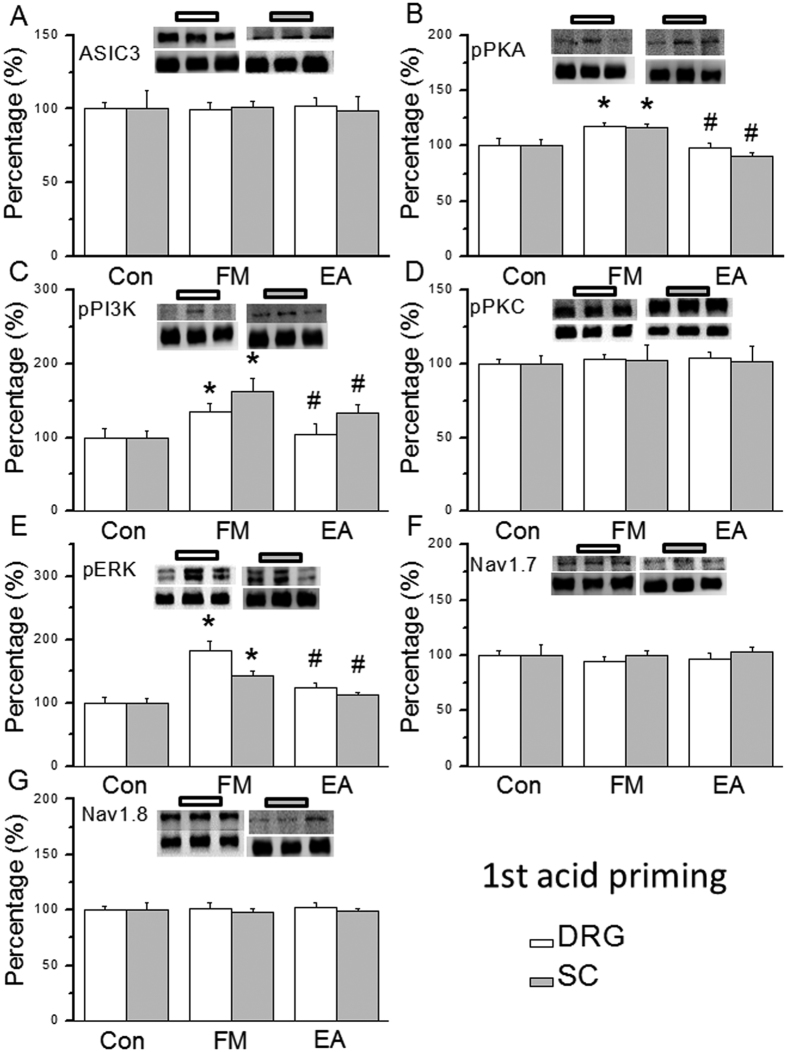
Expression levels of ASIC3-associated signaling pathway proteins in DRG and SC after first acid injection. (**A**) ASIC3, (**B**) pPKA, (**C**) pPI3K, (**D**) pPKC, (**E**) pERK, (**F**) Nav1.7, and (**G**) Nav1.8 expression levels in tissues from the Con, FM, EA groups (from left to right). Con = Control; FM = Fibromyalgia group; EA = Electroacupuncture. *p < 0.05 compared with the Con group. ^#^*p* < 0.05 compared with the FM group. The western blot bands at the top show the cropped target protein. The lower bands are cropped internal controls (β-actin or α-tubulin).

**Figure 3:**
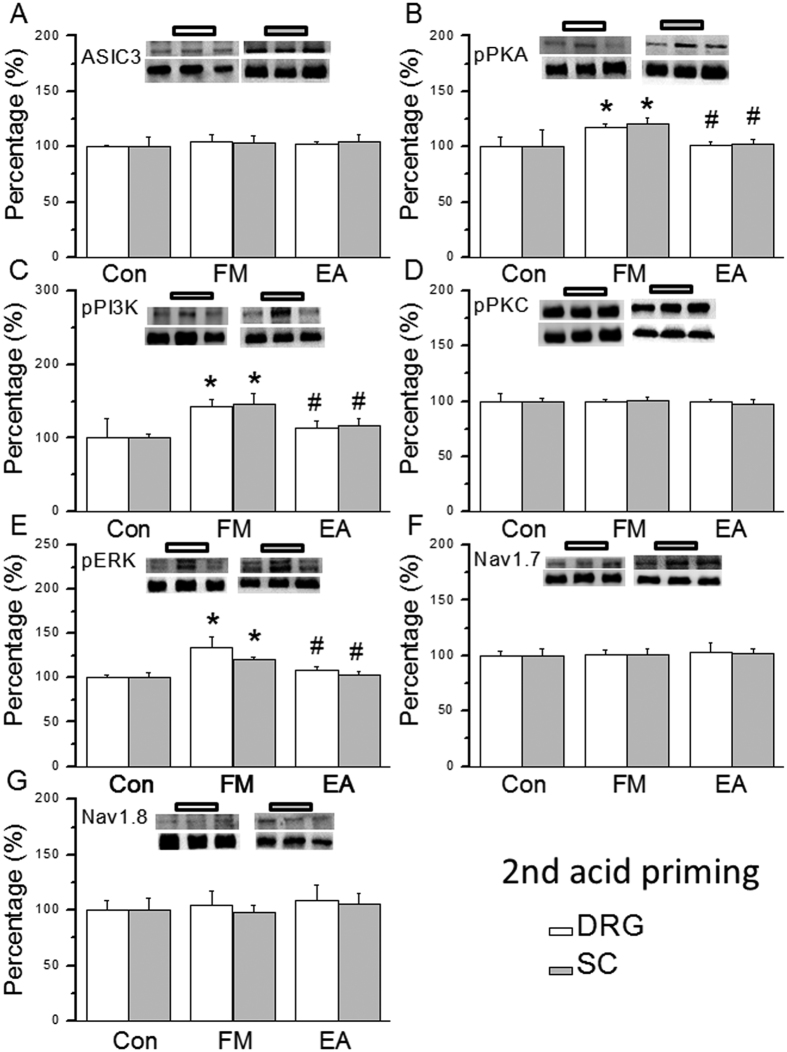
Expression levels of ASIC3-associated signaling pathway proteins in DRG and SC after second acid injection. (**A**) ASIC3, (**B**) pPKA, (**C**) pPI3K, (**D**) pPKC, (**E**) pERK, (**F**) Nav1.7, and (**G**) Nav1.8 expression levels in tissues from the Con, FM, EA groups (from left to right). Con = Control; FM = Fibromyalgia group; EA = Electroacupuncture. *p < 0.05 compared with the Con group. ^#^*p* < 0.05 compared with the FM group. The western blot bands at the top show the cropped target protein. The lower bands are cropped internal controls (β-actin or α-tubulin).

Much is known about the mechanical hyperalgesia induced by dual acidic saline injections; however, there is no evidence on the detailed mechanisms involved in the transient acid priming period. Thus, we identified which proteins are altered during the first and second acidic saline injections. We showed that ASIC3 was not altered in the DRG 15 min after the first saline injection (Fig. 2A, 99.8 ± 4.7%, *p* > 0.05, n = 6) or after EA treatment (Fig. 2A, 102.1 ± 5.5%, *p* > 0.05, n = 6). Interestingly, pPKA expression was increased within this window in FM mice (Fig. 2B, 117.4 ± 2.9%, *p* < 0.05 compared with Con group, n = 6) and rescued by EA (Fig. 2B, 98.0 ± 4.9%, *p* < 0.05 compared with FM group, n = 6). A similar pattern was observed for pPI3K signals (Fig. 2C, all *p* < 0.05, n = 6). There were no significant differences in pPKC among the 3 groups (Fig. 2D, all *p* > 0.05, n = 6). pERK levels increased after the first acidic saline injection (Fig. 2E, 181.5 ± 15.9%, *p* < 0.05 compared with Con group, n = 6), which was attenuated by EA treatment (Fig. 2E, 124.9 ± 5.8%, *p* < 0.05 compared with FM group, n = 6). Both Nav1.7 and Nav1.8 were not affected in any group (Fig. 2F and G, all *p* > 0.05, n = 6). Next, we assessed the central SC after the first saline injection. ASIC3 was not altered by FM (Fig. 2A, 101.3 ± 3.6%, p > 0.05, n = 6) or EA treatment (Fig. 2A, 98.9 ± 9.8%, p > 0.05, n = 6). Expression of pPKA was transiently increased in FM mice (Fig. 2B, 117.2 ± 2.5%, *p* < 0.05 compared with Con group, n = 6), and this effect was blocked by EA (Fig. 2B, 98.8 ± 2.9%, *p* < 0.05 compared with Con group, n = 6). The same results were observed for pPI3K (Fig. 2C, all *p* < 0.05, n = 6). There were no significant differences in pPKC levels among the 3 groups (Fig. 2D, all *p* > 0.05, n = 6). The expression of pERK was increased in the SC after the first acidic saline injection (Fig. 2E, 143.2 ± 7.4%, *p* < 0.05 compared with Con group, n = 6), which was alleviated by EA treatment (Fig. 2E, 112.5 ± 3.2%, *p* < 0.05 compared with FM group, n = 6). Both Nav1.7 and Nav1.8 levels were similar across all groups (Fig. 2F and G, all *p* > 0.05, n = 6). We examined if similar mechanisms occurred after a second acidic saline injection. ASIC3 was not changed at 15 min after acid injection in the DRG or SC of FM mice (Fig. 3A, all *p* > 0.05, n = 6). The FM-induced increase in pPKA was attenuated by EA (Fig. 3B, all *p* < 0.05, n = 6). A similar result was obtained for pPI3K (Fig. 3C, all p < 0.05, n = 6) but not for pPKC (Fig. 3D, all *p* > 0.05, n = 6). No changes in pERK (Fig. 3E, all *p* > 0.05, n = 6) or nociceptive Nav1.7 and Nav1.8 (Fig. 3F and G, all p > 0.05, n = 6) were found. These results provide a novel and detailed mechanism for FM priming.

### EA attenuates ASIC3-related nociceptive signals upregulated in DRGs 8 days after FM induction via opioid and adenosine pathways

**Figure 4:**
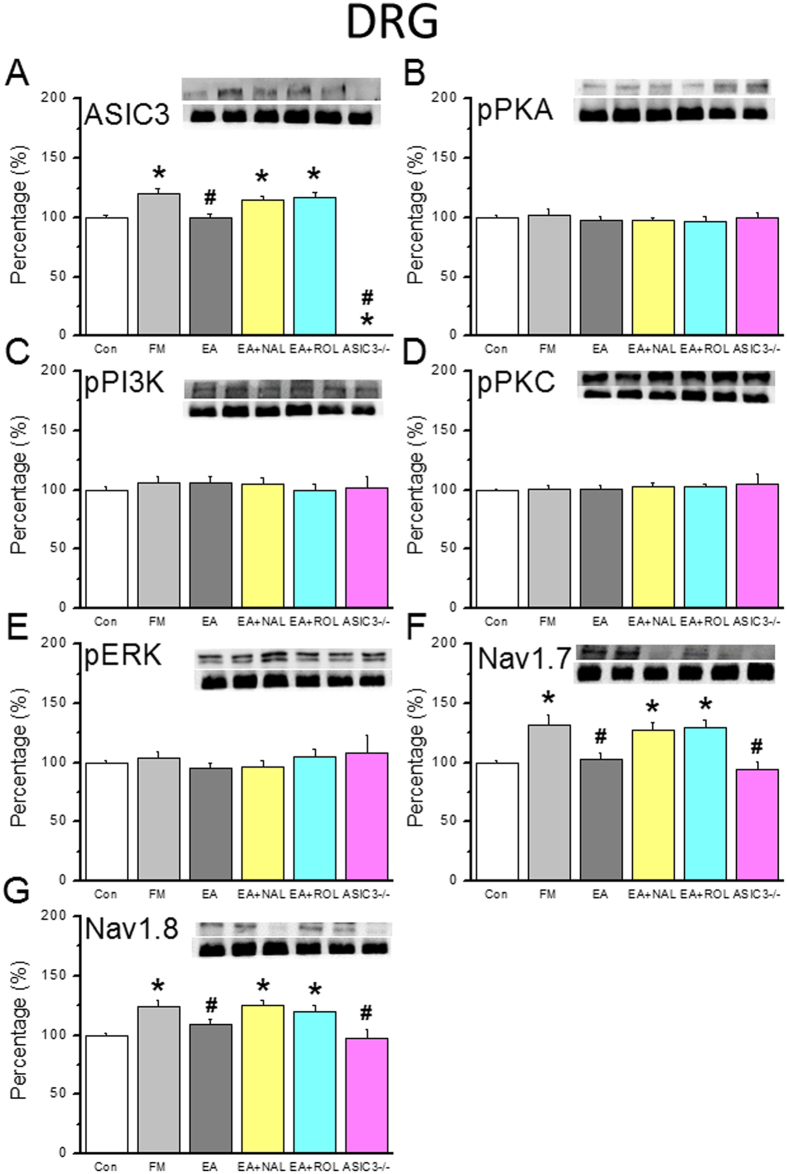
Expression levels of ASIC3-associated signaling pathway proteins in L3–5 DRG. (**A**) ASIC3, (**B**) pPKA, (**C**) pPI3K, (**D**) pPKC, (**E**) pERK, (**F**) Nav1.7, and (**G**) Nav1.8 expression levels in tissues from the Con, FM, EA, Nal, Rol, and ASIC3 null groups (from left to right). Con = Control; FM = Fibromyalgia group; EA = Electroacupuncture; Nal = Naloxone group; Rol = Rolofyllin group. ASIC3 null = ASIC3 gene deletion group. *p < 0.05 compared with the Con group. ^#^*p* < 0.05 compared with the FM group. The western blot bands at the top show the cropped target protein. The lower bands are cropped internal controls (β-actin or α-tubulin).

We examined if ASIC3-related nociceptive proteins were altered in our treatment model. ASIC3 showed a normal distribution in the control group (Fig. 4A, 100.1 ± 2.0%, n = 6) and was upregulated at day 8 after FM induction (Fig. 4A, 120.0 ± 4.6%, *p* < 0.05 compared with Con group, n = 6). EA treatment restored ASIC3 levels (Fig. 4A, 100.2 ± 2.9%, *p* < 0.05 compared with FM group, n = 6). Interestingly, these EA-mediated effects were blocked by naloxone (Fig. 4A, 114.8 ± 3.2%, *p* < 0.05 compared with Con group, n = 6), rolofylline (Fig. 4A, 116.4 ± 5.0%, *p* < 0.05 compared with Con group, n = 6), or not existed in ASIC3^−/−^ mice (Fig. 4A, 1.2 ± 0.3%, *p* < 0.05 compared with Con group, n = 6). Next, we assessed ASIC3-related downstream nociceptive signaling. Western blot results showed that the levels of pPKA, pPI3K, and pPKC were unchanged in all groups (Fig. 4B–D, all *p* > 0.05 compared with Con group, n = 6); thus, these molecules are not involved at the time point tested. Based on our previous results, we assessed if pERK-positive neurons were altered^7^. However, we found that pERK levels were similar in all groups (Fig. 4E, all *p* > 0.05, n = 6). We next examined nociceptive-relevant Nav channels. Nav1.7 was increased in FM mice (Fig. 4F, 132.2 ± 7.7%, *p* < 0.05 compared with Con group, n = 6) and restored to normal levels in the EA group (Fig. 4F, 102.7 ± 5.5%, *p* < 0.05 compared with FM group, n = 6). The EA-mediated Nav1.7 reduction was blocked by opioid, adenosine antagonists, or ASIC3 gene deletion (Fig. 4F, 94.8 ± 5.8%, *p* > 0.05 compared with Con group, n = 6). Similar effects on Nav1.8 protein levels were found (Fig. 4G, 129.2 ± 7.0%, *p* < 0.05 compared with Con group, n = 6). Therefore, we speculate that the ASIC3-Nav1.7/Nav1.8 signaling pathway is responsible for persistent FM-induced hyperalgesia in the peripheral DRG.

### EA attenuates the upregulation of ASIC3, Nav1.7, and Nav1.8 in the SC of FM mice via opioid and adenosine pathways

**Figure 5:**
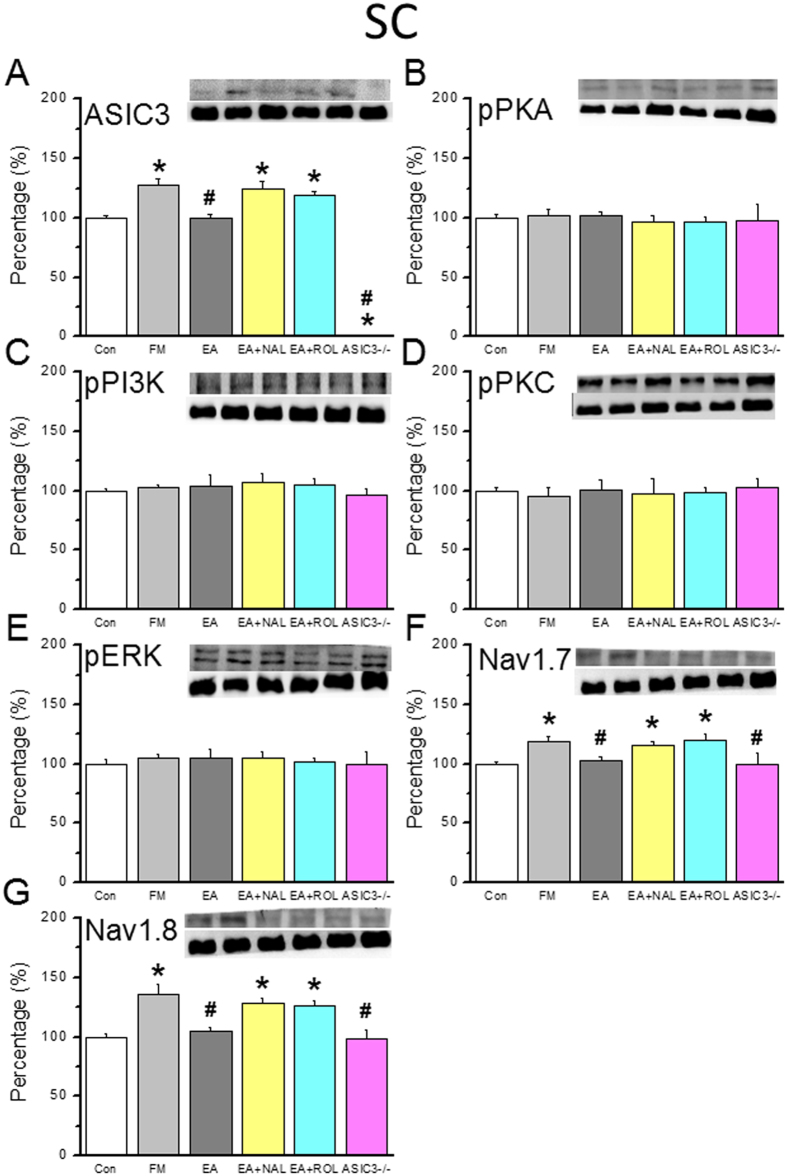
Expression levels of ASIC3-associated signaling pathway proteins in lumbar SC. (**A**) ASIC3, (**B**) pPKA, (**C**) pPI3K, (**D**) pPKC, (**E**) pERK, (**F**) Nav1.7, and (**G**) Nav1.8 expression levels in tissues from the Con, FM, EA, Nal, Rol, and ASIC3 null groups (from left to right). Con = Control; FM = Fibromyalgia group; EA = Electroacupuncture; Nal = Naloxone group; Rol = Rolofyllin group. ASIC3 null = ASIC3 gene deletion group (from left to right). Con = Control; FM = Fibromyalgia group; EA = Electroacupuncture; Nal = Naloxone group; Rol = Rolofyllin group. *p < 0.05 compared with the Con group. ^#^*p* < 0.05 compared with the FM group. The western blot bands at the top show the cropped target protein. The lower bands are cropped internal controls (β-actin or α-tubulin).

ASIC3 was distributed in the SC of control mice (Fig. 5A, 100.6 ± 0.4%, n = 6). After 8 days, FM mice showed an increase in ASIC3 at day 8 after FM induction (Fig. 5A, 127.9 ± 4.9%, *p* < 0.05 compared with Con group, n = 6). This increase was alleviated by 2 Hz EA stimulation at acupoint ST36 (Fig. 5A, 99.6 ± 3.5%, *p* < 0.05 compared with FM group, n = 6). Furthermore, naloxone (Fig. 5A, 125.0 ± 5.9%, *p* < 0.05 compared with Con group, n = 6) and rolofylline (Fig. 5A, 119.4 ± 3.0%, *p* < 0.05 compared with Con group, n = 6) blocked the effect of EA on ASIC3, and not existed in ASIC3^−/−^ mice (Fig. 5A, 0.9 ± 0.2%, *p* < 0.05 compared with Con group, n = 6); thus, EA acts through opioid and adenosine receptors. In addition, we assessed the levels of protein kinases, which are involved in several pathways. Proteins pPKA, pPI3K, and pPKC showed no change in the SC of FM mice on Day 8 after modeling (Fig. 5B–D, all *p* > 0.05, n = 6). Downstream of protein kinase, pERK was also unaltered on Day 8 (Fig. 5E, p > 0.05, n = 6). Thus, all of the tested kinases showed similar levels across all groups. By contrast, we determined that Nav1.7 was potentiated in the SC of mice on Day 8 after FM induction (Fig. 5F, 119.2 ± 3.9%, *p* < 0.05 compared with Con group, n = 6). EA treatment restored Nav1.7 levels in FM mice (Fig. 5F, 102.7 ± 3.8%, *p* < 0.05 compared with FM group, n = 6), and this effect was blocked by naloxone (Fig. 5F, 115.7 ± 3.3%, *p* < 0.05 compared with Con group, n = 6), rolofylline (Fig. 5F, 120.1 ± 5.0%, *p* < 0.05 compared with Con group, n = 6), and ASIC3 gene deletion (Fig. 5F, 99.7 ± 9.1%, *p* > 0.05 compared with Con group, n = 6). Nav1.8 is crucial for FM development; therefore, we assessed if EA altered Nav1.8 expression. Nav1.8 protein signals were distributed in DRG neurons (Fig. 5G, 99.9 ± 0.3%, n = 6) and augmented by FM modeling on Day 8 (Fig. 5G, 135.3 ± 9.0%, *p* < 0.05 compared with Con group, n = 6). Nav1.8 levels were ameliorated by EA at 2 Hz, suggesting that EA alleviates FM-mediated nociception (Fig. 5G, 104.4 ± 3.8%, *p* < 0.05 compared with FM group, n = 6). The effects of EA were blocked by naloxone (Fig. 5G, 127.7 ± 4.5%, *p* < 0.05 compared with Con group, n = 6), rolofylline (Fig. 5G, 126.6 ± 4.1%, *p* < 0.05 compared with Con group, n = 6), or ASIC3 gene deletion (Fig. 5G, 98.3 ± 7.8%, *p* > 0.05 compared with Con group, n = 6). These data support the hypothesis that EA acts via opioid and adenosine pathways in the SC.

### EA blocks the increase in ASIC3, pPKA, pPI3K, and pERK signaling pathway in the thalamus of FM mice via opioid and adenosine pathways

**Figure 6:**
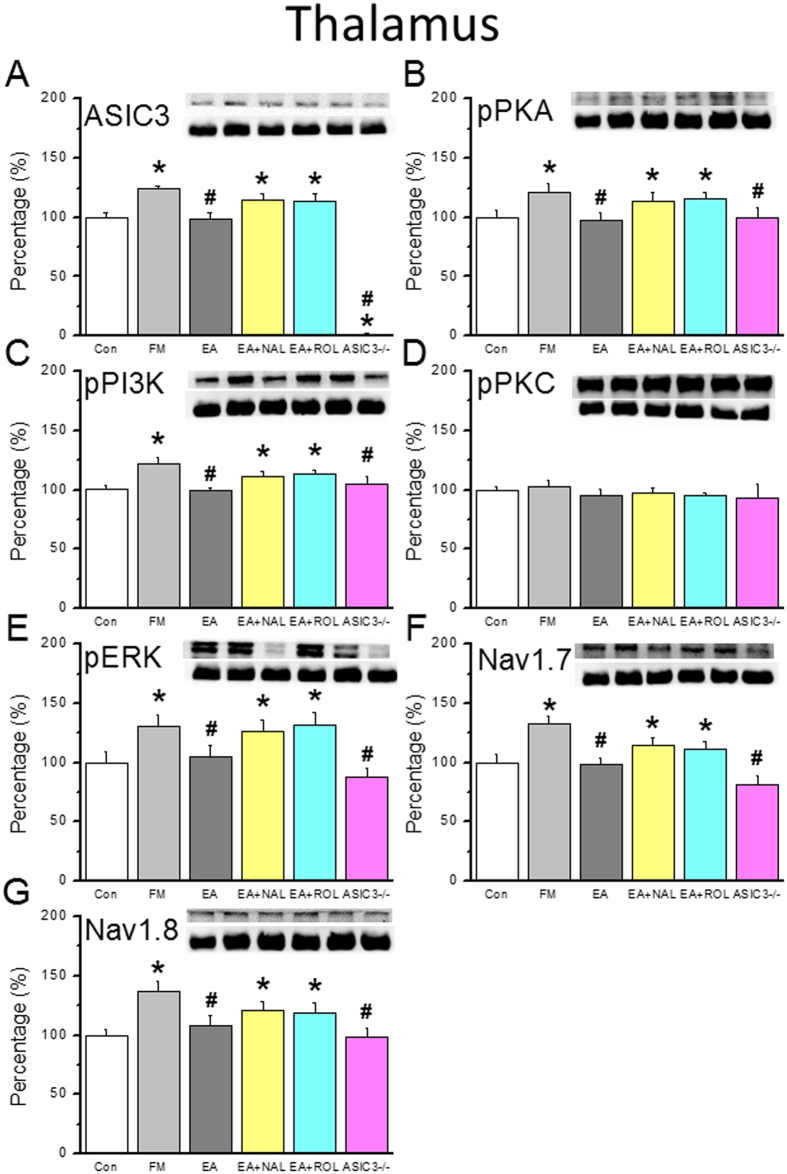
Expression levels of ASIC3-associated signaling pathway proteins in thalamus. (**A**) ASIC3, (**B**) pPKA, (**C**) pPI3K, (**D**) pPKC, (**E**) pERK, (**F**) Nav1.7, and (**G**) Nav1.8 expression levels in tissues from the Con, FM, EA, Nal, Rol, and ASIC3 null groups (from left to right). Con = Control; FM = Fibromyalgia group; EA = Electroacupuncture; Nal = Naloxone group; Rol = Rolofyllin group. ASIC3 null = ASIC3 gene deletion group (from left to right). Con = Control; FM = Fibromyalgia group; EA = Electroacupuncture; Nal = Naloxone group; Rol = Rolofyllin group. *p < 0.05 compared with the Con group. ^#^*p* < 0.05 compared with the FM group. The western blot bands at the top show the cropped target protein. The lower bands are cropped internal controls (β-actin or α-tubulin).

ASIC3 was normally distributed in the thalamus of control mice (Fig. 6A, 100.0 ± 4.5%, n = 6), increased in FM mice at day 8 after FM induction (Fig. 6A, 124.1 ± 2.9%, *p* < 0.05 compared with Con group, n = 6), and restored by EA (Fig. 6A, 98.4 ± 5.8%, *p* < 0.05 compared with FM group, n = 6). The rescue effect of EA on ASIC3 was mediated by opioid (Fig. 6A, 114.6 ± 5.2%, *p* < 0.05 compared with Con group, n = 6), adenosine pathways (Fig. 6A, 113.9 ± 6.2%, *p* < 0.05 compared with Con group, n = 6), or not existed in ASIC3^−/−^ mice (Fig. 6A, 1.2 ± 0.7%, *p* < 0.05 compared with Con group, n = 6). An increase of pPKA was found in the thalamus of FM mice (Fig. 6B, 121.5 ± 7.4%, *p* < 0.05 compared with Con group, n = 6) and rescued by EA (Fig. 6B, 97.5 ± 6.5%, *p* < 0.05 compared with Con group, n = 6). The effect of EA was blocked by opioid (Fig. 6B, 114.3 ± 6.6%, *p* < 0.05 compared with Con group, n = 6), adenosine (Fig. 6B, 115.5 ± 5.5%, *p* < 0.05 compared with Con group, n = 6) antagonists, or ASIC3 gene deletion (Fig. 6B, 99.5 ± 8.4%, *p* > 0.05 compared with Con group, n = 6) Similar data were obtained for pPI3K (Fig. 6C, n = 6) but not for pPKC (Fig. 6D, all p > 0.05, n = 6). pERK was increased in the thalamus of FM mice, which is consistent with a previous report^33^. The potentiation of pERK was reversed by EA via opioid and adenosine pathways (Fig. 6E, n = 6). The potentiation of pERK was also reversed in ASIC3^−/−^ mice (Fig. 6E, 99.5 ± 8.4%, *p* > 0.05 compared with Con group, n = 6). Nav1.7 was increased in the thalamus of FM mice (Fig. 6F, 132.5 ± 6.4%, *p* < 0.05 compared with Con group, n = 6) and rescued by EA (Fig. 6F, 98.2 ± 5.9%, *p* < 0.05 compared with FM group, n = 6) via opioid (Fig. 6F, 114.4 ± 6.3%, *p* < 0.05 compared with Con group, n = 6), adenosine (Fig. 6F, 111.3 ± 6.0%, *p* < 0.05 compared with Con group, n = 6), and ASIC3 pathways (Fig. 6F, 81.8 ± 7.2%, *p* > 0.05 compared with Con group, n = 6). A similar pattern was found for Nav1.8 (Fig. 6G, n = 6). These results indicate that ASIC3 in the thalamus is crucial for mechanical hyperalgesia in the FM mouse model.

### ASIC3, Nav1.7, and Nav1.8 immunoreactive signals were increased on Day 8 after FM induction and further attenuated by EA using immunohistochemical staining

**Figure 7:**
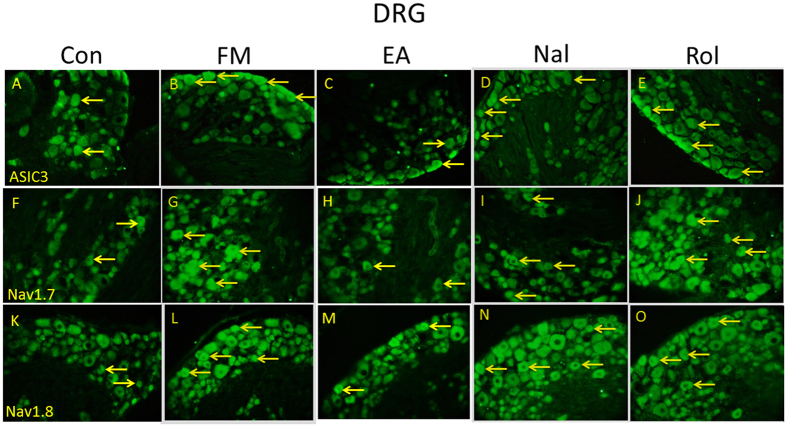
Expressions of ASIC3, Nav1.7, and Nav1.8 in the DRG of Con, FM, EA, Nal, and Rol mice. ASIC3-positive neurons (green) in the DRG of (**A**) Con, (**B**) FM, (**C**) EA, (**D**) Nal, and (**E**) Rol mice. Nav1.7-positive neurons (green) in the DRG of (**F**) Con, (**G**) FM, (**H**) EA, (**I**) Nal, and (**J**) Rol mice. Nav1.8-positive neurons (green) in the DRG of (**K**) Con, (**L**) FM, (**M**) EA, (**N**) Nal, and (**O**) Rol mice. Scale bar means 100 μm. Arrows identify immunopositive neurons.

Immunohistochemical labeling visualized by green fluorescence indicated that ASIC3 was existed in DRG neurons, dramatically increased 8 days after FM induction, and further reversed by EA (Fig. 7A–C). The effect of EA was reduced by naloxone and rolofylline (Fig. 7D and E). Furthermore, DRG neurons showed Nav1.7-positive signals in control mice, increased in FM, and further reversed by EA (Fig. 7F–H). The effect of EA was reversed by naloxone and rolofylline (Fig. 7I and J). Nociceptive Nav1.8-positive DRG neurons were normally distributed in control mice, potentiated in FM, and reversed by EA (Fig. 7K–M). The phenomenon was abolished in naloxone and rolofylline groups (Fig. 7N and O).

### Voltage-gated sodium currents in DRG neurons

**Figure 8:**
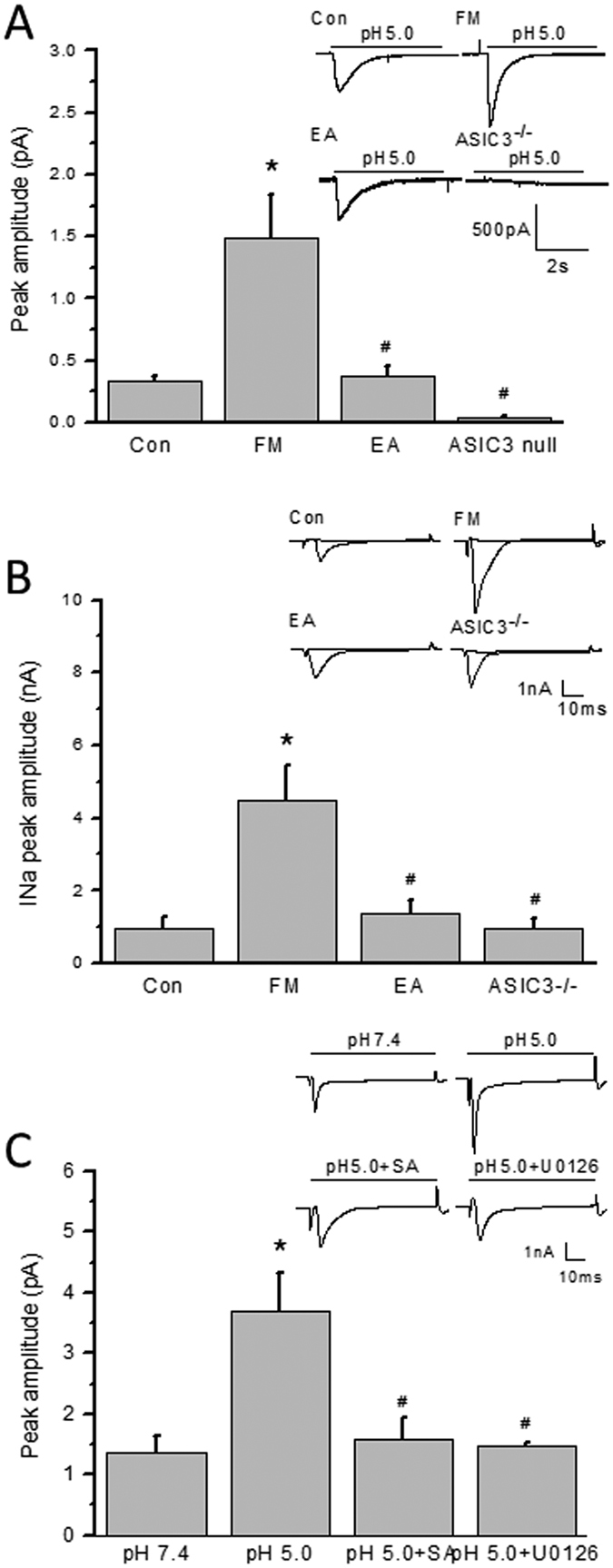
Acid-sensing ion channel 3 or voltage-gated sodium currents in L3-L5 DRG neurons. (**A**) Representative acid-sensing ion channel 3 currents traces in Con, FM, EA, and ASIC3^−/−^ groups. The acid-sensing ion channel 3 currents were induced by injection of pH5.0 saline directly to DRG neurons. (**B**) Representative voltage-gated sodium currents traces in Con, FM, EA, and ASIC3^−/−^ groups. The voltage-gated sodium currents were induced by membrane depolarization from −70 to 0 mV. (**C**) Representative voltage-gated sodium currents traces in pH 7.4, pH 5.0, pH 5.0 + SA, and pH 5.0 + U0126 groups. (**A**) Mean peak amplitudes of acid-sensing ion channel 3 currents in Con, FM, EA, and ASIC3^−/−^ groups. (**B**) Mean peak amplitudes of voltage-gated sodium currents in Con, FM, EA, and ASIC3^−/−^ groups. (**C**) Mean peak amplitudes of voltage-gated sodium currents traces in pH 7.4, pH 5.0, pH 5.0 + SA, and pH 5.0 + U0126 groups. *p < 0.05 compared with Con or pH 7.4 groups. ^#^*p* < 0.05 compared with FM or pH 5.0 groups.

To investigate whether voltage-gated sodium currents were affected by acid saline injection produced FM mice, we conducted whole-cell patch recording to measure the ASIC3 inward currents or voltage-gated sodium currents. In DRG neurons, acid-induced ASIC3 currents (Fig. 8A) or voltage-gated sodium currents (Fig. 8B) were significantly increased in DRG neurons 8 days after FM induction and further reversed by EA and ASIC3 gene deletion (Fig. 8A and B). Furthermore, acid saline (pH 5.0) significantly potentiated the voltage-gated sodium currents in control DRG (Fig. 8C) that can be abolished by ASIC3 blocker salicylic acid (SA) or ERK antagonist U0126 (Fig. 8C). All data were analyzed and plotted in Fig. 8A–C. These results provide evidences that acid could increase the amplitudes of voltage-gated sodium currents through ASIC3 and ERK pathways.

## Discussion

ASIC3 is involved in several types of pain syndromes such as inflammation and FM, that are highly associated with lower local pH^13^,^15^. These conditions activate ASIC3 via low pH, which initiates a transient inward current followed by a sustained inward current. The sustained currents from ASIC3 significantly prolong the sensation of acidosis pain in FM, arthritis, and inflammatory pain^46^,^47^. In this study, dual acid injections significantly initiated mechanical hyperalgesia via ASIC3, Nav1.7, and Nav1.8 signaling in both the peripheral DRG and the central SC. The potentiated ASIC3 signaling may respond to mechanical hyperalgesia from the peripheral acidosis site and transduce the acid-related pain signaling. Jeong *et al*. reported that ASIC3 was increased in the dorsal horn of the spinal cord in a spinal nerve ligation model, and this effect was ameliorated by amiloride, an ASIC3 blocker^48^.

Injections of the non-specific ASIC blocker amiloride, specific ASIC3 blocker APETx2, and artificial miRNA attenuated mechanical hyperalgesia in mice^49^,^50^,^51^. Izuma *et al*. demonstrated that ASIC3 in knee joint afferents was dramatically increased in an osteoarthritic mouse model. Injection of APETx2 reliably inhibited ASIC3 potentiation and pain behaviors^52^. Furthermore, in ASIC3^−/−^ mice, mechanical hyperalgesia after the induction of muscle inflammation was abolished^53^. Our previous results showed that intraplantar inflammation-mediated mechanical hyperalgesia was attenuated in ASIC3^−/−^ mice^54^. Repeated acidic saline injection-induced FM pain was not observed in ASIC3^−/−^ but was found in ASIC1^−/−^ mice, highlighting the crucial role of ASIC3 in this model^21^. A recent study showed that both the mechanical and thermal hyperalgesia initiated by two acidic saline injections were significantly reversed by 15 and 100 Hz EA^55^. Here we further determined that the EA-mediated attenuation of mechanical hyperalgesia was caused by a reduction in ASIC3, Nav1.7, and Nav1.8 proteins in both the peripheral DRG and central SC.

The Nav1.8 sodium channel was increased in rat and mouse DRG neurons after carrageenan and CFA-induced inflammatory pain^24^,^56^. Laird *et al*. showed that visceral pain and referred hyperalgesia were abolished in Nav1.8-null mice^57^. Intrathecal Nav1.8 antisense injections blocked Nav1.8 currents and attenuated mechanical allodynia after an intraplantar CFA injection^58^. Recently, A-803467, a novel specific blocker for Nav1.8, reduced nociception in animal models of neuropathic and inflammatory pain^59^. Our previous study showed that EA attenuated inflammatory pain by reducing Nav1.8 protein expression and functional currents^60^. Nielsen *et al*. reported that the sodium channel blocker mexiletine reliably reduce nociception of repeated injections of acidic saline^38^. We determined that EA attenuated mechanical hyperalgesia in FM mice by reducing ASIC3, Nav1.7, and Nav1.8 protein overexpression.

## Conclusion

In this study, EA at acupoint ST36 reliably reduced mechanical hyperalgesia and motor dysfunction in acidic saline injection-induced FM mice. Both nociceptive behavior and priming molecules were abolished in ASIC3-null mice, highlighting the crucial role of this protein in FM hyperalgesia. The pPKA, pPI3K, and pERK signaling pathway was potentiated in the DRG and SC during acid injection-induced hyperalgesia priming. ASIC3, Nav1.7, and Nav1.8 proteins were increased 8 days after FM modeling, and this effect was attenuated in the DRG and SC of FM mice by EA at acupoint ST36. Furthermore, the ASIC, pPKA, pPI3K, pERK, and Nav signaling pathway was increased in the thalamus, and this effect was attenuated by EA. A similar pattern was observed for pERK. We also provide physiological evidences that voltage-gated sodium currents were increased in FM DRGs and reduced in EA or ASIC3^−/−^ mice. Acid saline potentiated sodium currents through ASIC3 receptors and ERK pathway. Our results provide highly valuable data for the investigation of EA-related analgesic mechanisms and can be applied in clinical practice.

## Methods

### Animals

Experiments were conducted using C57/B6 mice (ages 8 to 12 weeks) purchased from BioLASCO Co. Ltd, Taipei, Taiwan. The sample size required for an alpha of 0.05 and a power of 80% was eight animals per group. After arrival, the mice were housed under a 12/12 h light/dark cycle, where water and food were available ad libitum. All of the procedures were approved by the Institute of Animal Care and Use Committee of China Medical University (permit No. 2016–061) and they were conducted in accordance with the Guide for the use of Laboratory Animals provided by the National Research Council and the ethical guidelines of the International Association for the Study of Pain. The number of animals used and their suffering were minimized.

### EA treatment and pharmacological injection

EA was applied using stainless steel needles (0.5″ inch, 32 G, YU KUANG, Taiwan) that were inserted into the muscle layer to a depth of 2–3 mm at ST36 acupoint. EA was administered immediate after the second injection acid saline every day at the same time (10:00–12:00 AM). A Trio-300 (Japan) stimulator delivered electrical square pulses for 15 min with a 100 μs duration and a 2 Hz frequency. The stimulation amplitude was 1 mA. For pharmacological injection, opioid or adenosine A1 receptor antagonist administration, **t**he opioid antagonist naloxone methiodide (Nal) (Sigma, St. Louis, MO, USA) in 100 μl of saline was injected i.p. at a dose of 10 mg/kg. The adenosine A1 receptor antagonist rolofylline (Ro) (Sigma, St. Louis, MO, USA) in 10 μl of saline was injected i.m. at a dose of 3 mg/kg into acupoint ST36. The opioid agonist endomorphin (EM) (Sigma, St. Louis, MO, USA), in 100 μl of saline, was administered intraperitoneally (i.p.) at a dose of 10 mg/kg once a day. Alternatively, the adenosine receptor agonist N6-cyclopentyladenosine (CPA) (Sigma, St. Louis, MO, USA) in 10 μl of saline was administered intramuscularly (i.m.) at a dose of 0.1 mg/kg into acupoint ST36 once a day. A dose of 20 pmole APETx2 (in 20 μL acid saline with a concentration of 1 μM APETx2) was injected into ST36 acupoint under light isoflurane anesthesia (1%).

### FM induction and animal behavior of mechanical hyperalgesia

We injected 20 μL of pH 4.0 acid saline into gastrocnemius muscle (GM) while the mice were anesthetized with isoflurane (1%). The second acid saline injection was performed at day 5 after first injection to successfully induce FM mice. All experiments were performed at room temperature (approximately 25 °C) and the stimuli were applied only when the animals were calm but not sleeping or grooming. Mechanical sensitivity was measured by testing the force of responses to stimulation with three applications of electronic von Frey filaments (North Coast Medical, Gilroy, CA, USA). Mice were placed on a metal mesh and adapted to the new environment for at least 30 min. The mechanical hyperalgesia of the hindpaw was measured before, 4 h, 1, 5, 6, 8 days after modeling. The FM mice were further euthanized and the L3-L5 DRG neurons, lumbar SC, and brain thalamus were isolated for analysis.

### Rotarod

The mice were put on a rotating machine with different speeds and durations can be tested. When mice fall down, the sensor can record the falling latency. Mice were placed on an accelerating rotarod apparatus (MK-660D, Muromachi Kikai, Tokyo, Japan) for 12 trials (4 trials per day on 3 consecutive days; D1-D3) with 5-min intervals between trials. Each trial lasted for 60 s with a steady speed of 4 rpm. The latency of each mouse falling from the rod was recorded for each trial. On day 4, mice underwent 4 rpm and increase the speeds with a 3 rpm increase with a 10 s duration.

### Tissue sampling and western blot analysis

L3-L5 DRG, lumbar SC, and thalamus tissues were excised to extract proteins. The total proteins were prepared by homogenizing the DRG, SC, and thalamus in lysis buffer containing 50 mM Tris-HCl (pH 7.4), 250 mM NaCl, 1% NP-40, 5 mM EDTA, 50 mM NaF, 1 mM Na_3_VO_4_, 0.02% NaNO_3_, and 1 × protease inhibitor cocktail (AMRESCO). The extracted proteins (30 μg per sample according to the BCA protein assay) were subjected to 8% SDS-Tris glycine gel electrophoresis and transferred to a PVDF membrane. The membrane was blocked with 5% non-fat milk in TBS-T buffer (10 mM Tris pH 7.5, 100 mM NaCl, 0.1% Tween 20), incubated with the first antibody in TBS-T and 1% bovine serum albumin, and incubated for 1 h at room temperature. A peroxidase-conjugated anti-rabbit antibody (1:5000) was used as the secondary antibody. The bands were visualized using an enhanced chemiluminescencent substrate kit (PIERCE) with LAS-3000 Fujifilm (Fuji Photo Film Co. Ltd). If appropriate, the image intensities of specific bands were quantified with NIH ImageJ software (Bethesda, MD, USA). The protein ratios were obtained by dividing the target protein intensities by the intensity of α-tubulin in the same sample. The calculated ratios were then adjusted by dividing the ratios from the same comparison group relative to the control.

### Immunohistochemical staining

Mice were anesthetized with isoflurane and then perfused transcardially with 4% paraformaldehyde. The tissue samples were cryprotected with 30% sucrose. The tissues were cut to a thickness of 15 μm and were post-fixed briefly with 4% paraformaldehyde and then incubated with blocking solution containing 3% BSA, 0.1% Triton X-100, and 0.02% sodium azide in PBS for 2 h at room temperature. After blocking, the sections were incubated at 4 °C overnight with the primary antibodies prepared in blocking solution. The secondary antibody was goat anti-rabbit (1:500) antibody (Molecular Probes, Carlsbad, CA, USA). We incubated the slices with fluorescence-conjugated secondary antibodies or avidin-biotin horseradish peroxidase complex (1 h), washed them three times with 0.1 M Tris buffer (5 min each), and then developed them in diaminobenzidine tetrahydrochloride (1–2 min), before washing three times with 0.1 M Tris buffer (5 min each). Finally, the sections were incubated with 0.1 M Tris buffer to stop the reaction. The slides were mounted with cover slips and visualized by using a CKX41 microscope with an Olympus U-RFLT50 Power Supply Unit (Olympus, Tokyo, Japan).

### Electrophysiology

L3–L5 DRGs were isolated from mice at 8 days after FM injection. For ASIC3 currents recording (checked by SA inhibition), recording cells were superfused in artificial cerebrospinal fluid (ACSF) containing (in mM) 130 NaCl, 5 KCl, 1 MgCl_2_, 2 CaCl_2_, 10 glucose, and 20 Hepes, adjusted to pH 7.4 with NaOH. ACSF solutions were applied by use of gravity. The recording electrodes were filled with (in mM) 100 KCl, 2 Na_2_-ATP, 0.3 Na_3_-GTP, 10 EGTA, 5 MgCl_2_, and 40 Hepes, adjusted to pH 7.4 with KOH. The pH 5.0 ACSF was titrated by 2-[N-morpholino]ethanesulfonic acid (MES). For Nav current recording, the internal solution contained (in mM) 10 NaCl, 110 CsCl, 20 tetraethylammonium-Cl, 2.5 MgCl_2_, 5 EGTA, 3 Mg^2+^-ATP, and 5 HEPES, adjusted to pH 7.2 with CsOH. The external solution contained (in mM) 100 NaCl, 5 CsCl, 30 tetraethylammonium-Cl, 1.8 CaCl_2_, 1 MgCl_2_, 0.1 CdCl_2_, 25 glucose, 5 4-aminopyridine, and 5 HEPES, adjusted to pH 7.4 with HCl. Osmolarity was adjusted to 300 mosm. Voltage-gated sodium channel currents were evoked by a 50 ms test pulse from −70 to 0 mV. All recordings were obtained at room temperature (25 °C) and completed within 24 h after plating. Salicylic acid (SA) was prepared from a 1-M stock solution (in 100% ethanol) to a final concentration of 500 μM in ACSF. U0126 was from Tocris-Cookson (Bristol, UK).

### Statistical analysis

All of the data were expressed as the mean ± standard error. Significant differences between groups were tested using ANOVA, followed by a post hoc Tukey’s test. *p* < 0.05 was considered significantly different.

## Additional Information

**How to cite this article**: Yen, L.-T. *et al*. Targeting ASIC3 for Relieving Mice Fibromyalgia Pain: Roles of Electroacupuncture, Opioid, and Adenosine. *Sci. Rep.* **7**, 46663; doi: 10.1038/srep46663 (2017).

**Publisher's note:** Springer Nature remains neutral with regard to jurisdictional claims in published maps and institutional affiliations.
